# Two Models of the Development of Social Withdrawal and Social Anxiety in Childhood and Adolescence: Progress and Blind Spots

**DOI:** 10.3390/children9050734

**Published:** 2022-05-17

**Authors:** Heidi Gazelle

**Affiliations:** Human Development and Family Science, Florida State University, Tallahassee, FL 32306, USA; hgazelle@fsu.edu; Tel.: +1-(850)-644-5722

**Keywords:** social anxiety, social withdrawal, shyness, development, peer relations, parenting, childhood, adolescence

## Abstract

This commentary features a review of two recently reformulated models of the development of child and adolescent: (1) social withdrawal by Rubin and Chronis-Tuscano 2021, and (2) social anxiety by Spence and Rapee 2016. The articles that present these reformulated models now cover advances made during the prior 12 to 18 years of research, including increased knowledge of genetic vulnerability to anxiety and longitudinal patterns of development, and acknowledgement of multiple pathways towards and away from the development of social withdrawal or social anxiety (i.e., equifinality, multifinality). However, these reformulated models also contain several blind spots. The model of social withdrawal development would be improved by explicitly referring to peer treatment (not only attitudinal peer rejection), especially peer exclusion; and incorporating the potential development of clinically significant anxiety in childhood (not only adolescence) and delays in developmental milestones in adulthood. The model of social anxiety development would be improved by featuring social withdrawal as a proximal affective-behavioral profile (rather than a temperament) and drawing upon the literature on social withdrawal and its links to peer relations. Overall, there is a continuing lack of integration between developmental and clinical research and models of the development of social withdrawal and social anxiety.

## 1. Introduction

Conceptual models of the development of social withdrawal [1] and etiology of social anxiety [2] in childhood and adolescence have recently been updated by their authors to reflect the past 12 to 18 years of research. These updated models [3,4] reflect progress in the understanding of the development of social withdrawal and social anxiety, but also reveal blind spots in conceptualization. Furthermore, these models reveal a continuing lack of integration of developmental and clinical approaches to research on social withdrawal and social anxiety, despite earlier efforts towards integrating these research traditions [5,6].

Integrating developmental and clinical models and research on social withdrawal and social anxiety is important because it would foster a greater understanding of the developmental processes (e.g., parenting, peer relations, emotion regulation, social cognitive, and self-development processes) that lead to divergent psychosocial adjustment among vulnerable children and youth over time. Vulnerable children and youth can move toward (equifinality) or away from (multifinality [7]) the development of the clinical disorder and accompanying impairment, and encounter roadblocks to full participation in society (e.g., as manifested in delayed or unsuccessful attempts at adult developmental milestones for education, employment, romantic partnership, procreation, etc.). Likewise, clinical research on social cognitive patterns related to the development and maintenance of social anxiety [8] should inform investigations of the development of social withdrawal.

### Definitions

*Social withdrawal* is an umbrella term which identifies children and youths who remain alone at high rates relative to their age mates in social contexts (e.g., school recess) in which peers are available as interaction partners [3]. Social anxiety/shyness is the most common motivation for social withdrawal, although some children and youths demonstrate social withdrawal for other reasons (e.g., unsociability or lack of interest in peer interaction) [9,10,11]. Anxious solitary/withdrawn children and youths are conceptualized as wanting to interact with peers (possessing normative social approach motivation), but being held back by social anxiety (or elevated social avoidance motivation) that occurs even with familiar peers such as classmates [12]. Social withdrawal, which refers to a child’s solitary behavior, should not be confused with terms that refer to peer treatment of a child, such as peer exclusion or isolation by the peer group [12]. Rubin and Chronis-Tuscano’s [3] model posits that social withdrawal in childhood may forecast social anxiety in early adolescence and beyond.

The diagnosis of *social anxiety disorder* (SAD, previously known as “social phobia”) requires that an individual experiences fear or anxiety in social situations, avoids or endures social situations with anxiety, experiences these symptoms for at least six months, and that these symptoms are accompanied by distress or life impairment (e.g., interfere with attending childcare, school, or work) [4,13,14]. Importantly, in children and adolescents, anxiety must occur in interactions with peers (not just adults). Spence and Rapee’s model [4] of the etiology of social anxiety places social withdrawal in a category labelled “genes/temperament”.

## 2. Strengths and Blind Spots of Reformulated Models

### 2.1. Development of Social Withdrawal: Rubin and Chronis-Tuscano, 2021

A strength of Rubin and Chronis-Tuscano’s [3] model of the development of social withdrawal is its detailed description of the role of children’s and youths’ key interpersonal relations with parents and peers in contributing to the development of children and youths’ social withdrawal by developmental period, including infancy, toddlerhood, preschool, early elementary school, late elementary school, and early adolescence. The model also features additional influences on the development of social withdrawal in childhood and adolescence that are connected to children’s and youths’ relations with their parents and peers, including behaviorally inhibited toddler temperament, influences on parents, parenting beliefs, and sociological setting conditions.

Rubin and Chronis-Tuscano [3] deliver an insightful critique of the current state of the literature that informs their model of the development of social withdrawal. They note that evidence is modest or lacking for (1) linking behaviorally inhibited temperament among *unfamiliar* peers to social withdrawal among *familiar* peers, (2) *sub*-cultural differences in the prevalence and outcomes of social withdrawal, and (3) the development and consequences of social withdrawal in late adolescence and young adulthood. Additionally, they note that although the study of social motivations associated with social withdrawal has become popular [10], there is little evidence that these social motivations relate to observable behavior [9].

Although the description of the role of peer difficulties in maintaining and exacerbating anxious withdrawal in childhood and adolescence is a strength of Rubin and Chronis-Tuscano’s [3] model, in an unfortunate blind spot, the model refers to peer difficulties as “peer rejection.” Peer rejection is a construct that carries a specific meaning for peer relations researchers: peer rejection is an *attitudinal* variable that indicates that a child or youth is disliked by many of his or her peers [15]. Although peer attitudes and peer treatment of children and youths are often related, they are not one and the same. Children and youths are often directly affected by the way they are overtly treated by peers, rather than by covert peer attitudes. For instance, peer exclusion, or being left out of peer interaction, predicts not only the maintenance of anxious solitude/withdrawal over the course of middle childhood after controlling for peer rejection [12], but also the emergence of anxious solitude/withdrawal in early adolescence in youth with no previous history of anxious solitude/ withdrawal [16]. Ironically, explicit mention was made of peer exclusion in the earlier iteration of the model [1], but it is missing from the updated model [3]. Nonetheless, Rubin and Chronis-Tuscano draw upon some evidence of peer mistreatment in support of their model, but mislabeling this as evidence of peer rejection is not likely to accurately communicate these findings to the community of researchers who have a different shared understanding of the meaning of this term. Importantly, featuring peer rejection versus peer exclusion as a key interpersonal process in a model of the development of social withdrawal is not just a matter of semantics, but rather of accuracy in specifying the interpersonal processes (i.e., overt peer treatment) known to contribute to the development of social withdrawal in the current evidence base [12,16,17,18,19].

Rubin and Chronis-Tuscano’s model [3] is ultimately aimed at explaining the development of internalizing problems in socially withdrawn children and youth, which makes it comparable to Spence and Rapee’s model of the etiology of social anxiety [4]. Rubin and Chronis-Tuscano’s model [3] accurately suggests that childhood social withdrawal may lead to the development of anxiety, but in another blind spot (or perhaps understatement), proposes that this does not occur until the early adolescent period (12 years of age and beyond). However, even preschool age children can be diagnosed with anxiety disorders [20,21]. Moreover, of children identified as anxious solitary/withdrawn in fourth grade (at about 9 years of age), a third were also diagnosed with SAD [6]. A large study of prevalence [22] indicated that the age of onset for SAD occurred by 13 years of age for 50 percent of individuals who developed the disorder (the median), by eight years of age for 25 percent of individuals, and by 5 years of age for 5 percent of individuals. Importantly, these joint age of onset and prevalence rates for child and adolescent SAD were obtained retrospectively from individuals aged 18 and up in this large-scale prevalence study [22], and prospective studies of children have produced evidence of similar rates in unselected samples [21,23], and substantially higher rates in children at risk due to social withdrawal [6] or behavioral inhibition [24]. Thus, models of the development of social withdrawal should acknowledge the potential for clinically significant social anxiety to develop in middle childhood, and probably also in the preschool period. Research is needed on processes involved in the development of clinically significant social anxiety in withdrawn children and adolescents. Peer adversity may be a key factor contributing to the development of clinically significant social anxiety and other internalizing problems [12].

To address another blind spot, in addition to internalizing problems, the equally important potential adult outcomes of childhood social withdrawal—delays in achieving adult developmental milestones [25] (e.g., moving out of the parental home, obtaining a higher education, initiating a career, initiating a romantic partnership, cohabitation with a romantic partner, childbearing, childrearing, income, homeownership)—should be added to the model of the development of social withdrawal. Delays in adult developmental milestones are likely to have profound impacts on wellbeing. For instance, the absence of a romantic partner and children contributes to loneliness, which can compromise life satisfaction and even forecast premature death [26].

Consideration of these recommendations in developmental research and prevention efforts for social withdrawal in children and adolescents could yield improved cross-fertilization in developmental and clinical knowledge bases, as well as prevention and intervention efficacy for withdrawn children and adolescents. First, awareness of clinically significant anxiety could be increased in families, schools, and other professionals who serve children and adolescents to facilitate the early identification of young people suffering with anxiety and limit the extent that this anxiety may compromise healthy development by facilitating prevention and early treatment. Second, the overt forms of peer mistreatment (i.e., peer exclusion) that are most often detrimental to healthy development in withdrawn children could be targeted for prevention and intervention [27]. Third, patterns of cognition characteristic of social anxiety and its maintenance [4] could be investigated in socially withdrawn children and adolescents, and these cognitive patterns could also become the targets of prevention and intervention efforts for withdrawn children and adolescents. Fourth, prevention and intervention efforts for withdrawn children and adolescents could explicitly support the achievement of adult developmental milestones for participation in society (e.g., facilitate healthy friendships and romantic relationships, transitions to higher education, career counseling, financial counseling, and parenting skills).

### 2.2. Development of Social Anxiety: Spence and Rapee, 2016

Spence and Rapee’s model [4] posits that the interplay between “genes/temperament” and environmental factors contributes to proximal behavioral and cognitive factors, which in turn determine the individual’s level of anxiety. Additionally, both personal (age, gender) and cultural factors influence whether the individual’s anxiety engenders sufficient life impairment to warrant a diagnosis of SAD.

The literature review, which provides the basis for this updated model, has multiple strengths. For example, it covers research on genetic vulnerability to anxiety. This literature indicates that both genes (multiple genetic variants which individually have a small impact, but in combination have a significant impact) and *non-shared* environment (environmental influences which are *not* shared by family members) have a strong impact on anxiety. Major sources of non-shared environmental influences are children’s and youths’ peer relations and other experiences at school [28]. Also, Spence and Rapee [4] review research indicating that behaviorally inhibited toddler temperament, or wariness to unfamiliar people and situations/objects [29], conveys an increased risk of anxiety over the course of development. Additionally, Spence and Rapee [4] review research on attentional “bias” towards threat, and nuanced cognitive and social cognitive mechanisms implicated in social anxiety.

In their analysis of the extant literature, Spence and Rapee [4] also come to the critical conclusion that about fifteen percent of children who do *not* have a history of behaviorally inhibited temperament nonetheless develop SAD (equifinality). Although they do not elaborate on this important observation, it may be that non-shared environmental influences that take on the form of negative interpersonal learning processes, such as peer adversity and other experiences at school, also contribute to the development of social anxiety in children and youths who do *not* have early histories of behavioral inhibition or withdrawal. In support of this contention, the literature on social withdrawal provides evidence that peer exclusion predicts increasing anxious solitude in early adolescence, even among youths without a prior history of social withdrawal in childhood [16].

However, a major blind spot of Spence and Rapee’s model [4] is its treatment of social withdrawal. Social withdrawal is included under “genes/temperament” rather than as a “proximal behavior” in the figure depicting the model, and the literature review which supports the model does not draw upon the literature on social withdrawal. This treatment of social withdrawal implies that it is the same phenomenon as behaviorally inhibited temperament. This assumption is not warranted for several reasons. First, as noted by Rubin and Chronis-Tuscano [3], there is little empirical evidence for the link between behavioral inhibition to the unfamiliar and social withdrawal among familiar peers. Second, the conceptualization of the two constructs differs. Behavioral inhibition is conceptualized as wariness in the face of the unfamiliar (people, situations, objects), whereas withdrawal is conceptualized as elevated solitary behavior among peers due to social evaluative concerns (fears about being poorly treated by peers and/or not behaving competently with peers) [12]. Behavioral inhibition is believed to be rooted in a low threshold for stimulation in the brain’s limbic system [29], whereas social withdrawal is linked to negative interpersonal learning experiences with parents and peers [3]. Behavioral inhibition may increase the risk for the development of social withdrawal among familiar peers, but evidence suggests that social withdrawal develops in non-behaviorally inhibited children who have negative interpersonal learning experiences [30,31]. A great strength of the literature on the development of social withdrawal is a detailed assessment of key interpersonal learning mechanisms with parents and peers. Therefore, in not drawing upon this literature, models of social anxiety development fail to build upon evidence for the interpersonal learning processes that engender and/or exacerbate social anxiety and social avoidance.

Considerations of these recommendations in clinical prevention and early treatment for SAD in children and adolescents could yield many improvements in selection for intervention as well as intervention efficacy. First, at-risk children and adolescents could be targeted for intervention based on social withdrawal or the combination of social withdrawal and peer difficulties, not only behavioral inhibition. This may address the psychosocial needs and mental health of at-risk children and adolescents that are currently overlooked. Second, these children and adolescents’ peer relations (e.g., peer exclusion) could become a target of intervention [27], alongside efforts to ameliorate individual social and emotional competence. Third, interventions could be timed just prior to the beginning of the new school year to take advantage of the natural rhythms of this ecological transition to maximize children’s and adolescents’ chances for a fresh start when they have encountered peer difficulties [16]. This opens the possibility that naturally occurring interactions and relationships with peers could serve a therapeutic purpose. 

## 3. Conclusions

Rubin and Chronis-Tuscano and Spence and Rapee’s updated models [3,4] reflect progress in the understanding of the development of social withdrawal and social anxiety during the last 12–18 years, but also reveal blind spots in conceptualization and evidence, as well as continuing lack of integration of developmental and clinical research and conceptual models of social withdrawal and social anxiety, despite earlier efforts towards integrating these research traditions [5,6]. Research at the intersection of the development of social withdrawal and social anxiety has the potential to provide a more integrated explanation and evidence base for both phenomena by borrowing from the strengths of both traditions and highlighting cross-disciplinary gaps in evidence and intervention efforts.
